# Tetra­aqua­(2,2′-bipyridine-κ^2^ *N*,*N*′)nickel(II) sulfate

**DOI:** 10.1107/S160053681202538X

**Published:** 2012-06-16

**Authors:** Sujirat Boonlue, Chatphorn Theppitak, Kittipong Chainok

**Affiliations:** a Department of Chemistry, Faculty of Science, Naresuan University, Muang, Phitsanulok 65000, Thailand

## Abstract

The asymmetric unit of the title complex, [Ni(C_10_H_8_N_2_)(H_2_O)_4_]SO_4_, consists of a complex [Ni(bipy)(H_2_O)_4_]^2+^ cation (bipy = 2,2′-bipyridine) and a non-coordinating [SO_4_]^2−^ anion. The Ni^II^ atom is six-coordinated in a distorted octa­hedral geometry defined by the two N atoms of the bipy ligand and four water O atoms. The crystal structure contains extensive classical O—H⋯O hydrogen bonds, which link the ions into a two-dimensional array in the *ab* plane. Layers are connected into a three-dimensional supra­molecular structure by C—H⋯O inter­actions.

## Related literature

For the structures and properties of coordination complexes with bipy as a ligand, see: Graaf & Sousa (2010 ▶); Baruah *et al.* (2007 ▶); Schubert & Eschbaumer (2002 ▶); Harvey *et al.* (1999 ▶); Damrauer *et al.* (1997 ▶); Healy *et al.* (1984 ▶)
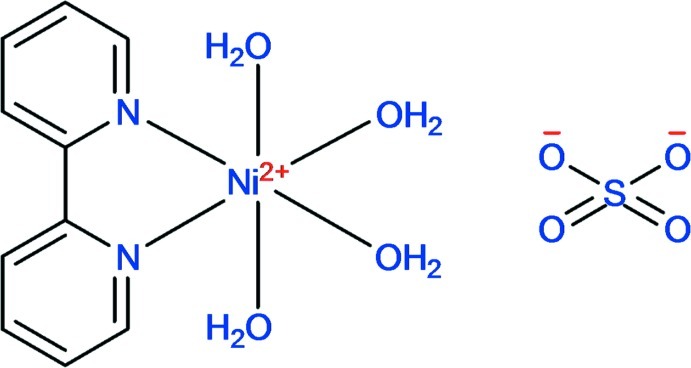


## Experimental

### 

#### Crystal data

[Ni(C_10_H_8_N_2_)(H_2_O)_4_]SO_4_
*M* *_r_* = 383.02
Orthorhombic, 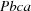

*a* = 12.3035 (7) Å
*b* = 11.6560 (7) Å
*c* = 20.7112 (10) Å
*V* = 2970.2 (3) Å^3^
*Z* = 8
Mo *K*α radiation
μ = 1.49 mm^−1^
*T* = 298 K
0.25 × 0.20 × 0.20 mm

#### Data collection

Bruker SMART APEX CCD area detector diffractometer
Absorption correction: multi-scan (*SADABS*; Bruker, 2003 ▶) *T*_min_ = 0.707, *T*_max_ = 0.755
11218 measured reflections
3626 independent reflections
3024 reflections with *I* > 2σ(*I*)
*R*_int_ = 0.045

#### Refinement

*R*[*F* ^2^ > 2σ(*F* ^2^)] = 0.039
*wR*(*F* ^2^) = 0.092
*S* = 1.07
3626 reflections
231 parameters
12 restraints
H atoms treated by a mixture of independent and constrained refinement
Δρ_max_ = 0.58 e Å^−3^
Δρ_min_ = −0.70 e Å^−3^

### 

Data collection: *SMART* (Bruker, 2001 ▶); cell refinement: *SAINT* (Bruker, 2002 ▶); data reduction: *SAINT*; program(s) used to solve structure: *SHELXS97* (Sheldrick, 2008 ▶); program(s) used to refine structure: *SHELXL97* (Sheldrick, 2008 ▶); molecular graphics: *ORTEP-3 for Windows* (Farrugia, 1997 ▶) and *DIAMOND* (Brandenburg, 2006 ▶); software used to prepare material for publication: *publCIF* (Westrip, 2010 ▶).

## Supplementary Material

Crystal structure: contains datablock(s) global, I. DOI: 10.1107/S160053681202538X/tk5105sup1.cif

Supplementary material file. DOI: 10.1107/S160053681202538X/tk5105Isup2.cdx

Structure factors: contains datablock(s) I. DOI: 10.1107/S160053681202538X/tk5105Isup3.hkl

Additional supplementary materials:  crystallographic information; 3D view; checkCIF report

## Figures and Tables

**Table 1:** Hydrogen-bond geometry (Å, °)

*D*—H⋯*A*	*D*—H	H⋯*A*	*D*⋯*A*	*D*—H⋯*A*
O1—H1*B*⋯O5	0.86 (2)	1.90 (2)	2.754 (2)	173 (3)
O1—H1*A*⋯O7^i^	0.86 (2)	1.83 (2)	2.683 (2)	173 (4)
O2—H2*B*⋯O5^ii^	0.86 (2)	1.87 (2)	2.728 (2)	174 (3)
O2—H2*A*⋯O8^iii^	0.86 (2)	1.97 (2)	2.800 (2)	162 (3)
O3—H3*B*⋯O6^iii^	0.86 (2)	1.89 (2)	2.736 (2)	168 (4)
O3—H3*A*⋯O8^i^	0.86 (2)	1.98 (2)	2.840 (2)	172 (3)
O4—H4*B*⋯O6	0.86 (2)	1.86 (2)	2.712 (2)	172 (3)
O4—H4*A*⋯O8^ii^	0.86 (2)	1.90 (2)	2.760 (2)	174 (3)
C8—H8⋯O6^iv^	0.93	2.55	3.310 (3)	139

Symmetry codes: (i) 
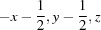
; (ii) 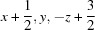; (iii) 
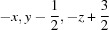
; (iv) 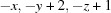.

**Fig. 1.**
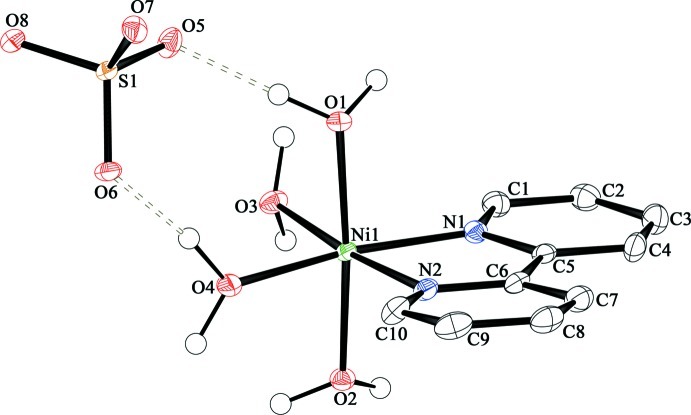
The asymmetric unit of I. Displacement ellipsoids are drawn at the 50% probability level.

**Fig. 2.**
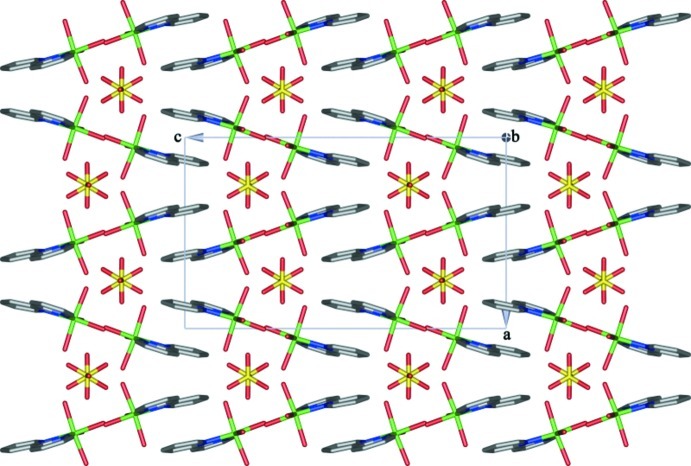
A view of the packing of I along the b axis.

**Fig. 3.**
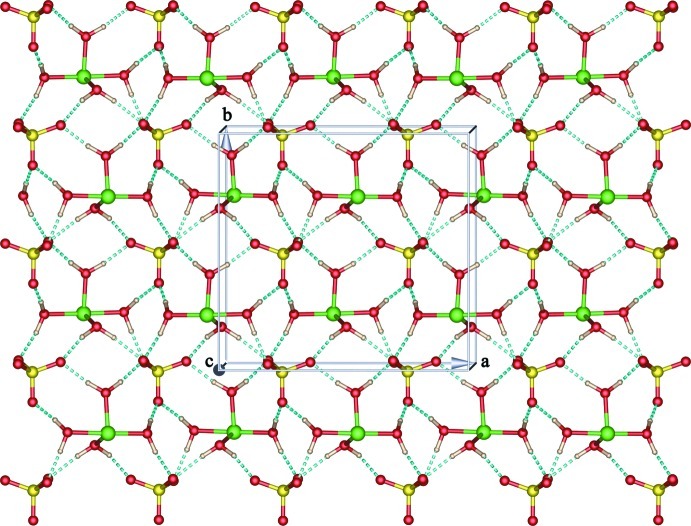
Two-dimensional sheet in the ab plane where ions are linked via classical O—H···O hydrogen bonds in I. Bipy molecules are omitted for clarity.

**Fig. 4.**
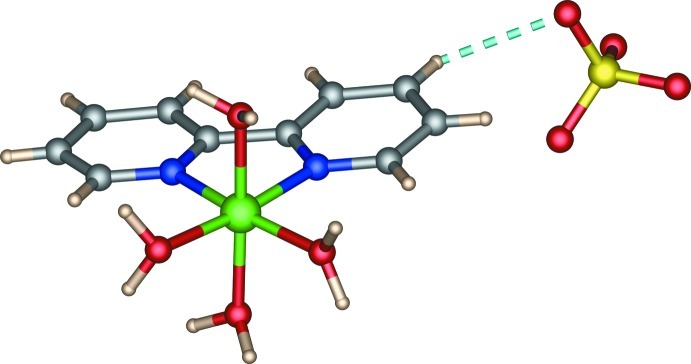
A view of the weak C—H···O hydrogen bond (a) in I. These serve to connect the layers into a three-dimensional architecture.

## supplementary crystallographic information

### Comment

2,2'-Bipyridine (bipy) is well known as a bidentate chelating ligand. It is one of the most widely used ligands in coordination and supramolecular chemistry. Nowadays, numerous transition metal complexes containing the bipy ligand are known in the literature. These include the mononuclear compound contains the [*M*(bipy)*_n_*]^2+^ core (*n* = 1–3). Some of these complexes were found to have interesting catalytic (Schubert & Eschbaumer 2002), magnetic (Graaf & Sousa 2010) and optic (Damrauer *et al.*, 1997) properties. Here, we report the crystal structure of title compound, I, a new member of [*M*(bipy)*_n_*]^2+^ family, which is isostructural to [Cd(bipy)(H_2_O)_4_]SO_4_ (Harvey *et al.*, 1999).

The asymmetric unit of I consists of the cationic complex [Ni(bipy)(H_2_O)_4_]^2+^ and an uncoordinated [SO_4_]^2-^ anion as shown in Fig. 1. The Ni^II^ atom displays a distorted octahedral environment. The two bipy N atoms and two water O atoms (O3 and O4) define an equatorial plane with a maximum deviation of -0.069 (1) Å for atom N2 and with the Ni1 atom lying 0.007 (1) Å out of the plane. The O atoms (O1 and O2) of the remaining two water molecules complete the octahedron in the axial positions, Table 1. The bipy ligand in I exhibits the usual acute N···N bite distances of 2.641 (2) Å [N1···N2]. The bite angle is 79.3 (1)° for N1—Ni1—N2. These are one of the main factors accounting for the distortion from the ideal octahedral geometry (90°) of the Ni^II^ centre. The mean Ni—N (2.069 (2) Å) and Ni—O (2.068 (2) Å) bond lengths are in agreement with those reported for other bipy complexes of nickel such as [Ni(bipy)(H_2_O)_4_]SO_4_.2H_2_O (Healy *et al.*, 1984) and [Ni(bipy)(H_2_O)_4_][C_12_H_8_O_8_] (Baruah *et al.*, 2007).

Fig. 2 shows the packing of I viewed along the *b* axis. An extensive classical O–H···O hydrogen bonds link the [Ni(bipy)(H_2_O)_4_]^2+^ cations to the [SO_4_]^2-^ anions forming a two dimensional sheet in the *ab* plane, Fig. 3 and Table 2. There are also C–H···O interactions involving the ligated bipy molecules and the [SO_4_]^2-^ anions, Fig. 4. The latter interactions link the two-dimensional sheets into a three-dimensional supramolecular network.

### Experimental

The title compound was obtained unexpectedly in an attempt to synthesize the cyanide-bridged bimetallic silver(I)-nickel(II) coordination polymers. In a typical experiment, K[Ag(CN)_2_] (40.1 mg, 0.2 mmol) was dissolved in 3 ml of DMF/MeCN, and this was pipetted into one side of the H-tube. NiSO_4_.6H_2_O (52.7 mg, 0.2 mmol) and bipy (31.1 mg, 0.2 mmol) were dissolved in 3 ml of DMF/MeCN, and this was pipetted into the other side arm of the H-tube. The H-tube was then carefully filled with distilled water. Upon slow diffusion for two weeks, blue block-shaped single crystals of I were formed in the silver-containing side of the H-tube. Yield: 25.8 mg (64% based on K[Ag(CN_2_]).

### Refinement

The C-bound hydrogen atoms were placed in the geometrically idealized positions based on chemical coordinations and constrained to ride on their parent atom positions with a C–H distances of 0.93 Å and with *U*_iso_(H) = 1.2*U*_eq_(C) for aromatic. The water-bound hydrogen atoms were located in a difference Fourier map and refined being in their as-found positions with the O—H distance = 0.86±0.01 Å.

### Crystal data

**Table:**

[Ni(C_10_H_8_N_2_)(H_2_O)_4_]SO_4_	*F*(000) = 1584
*M**_r_* = 383.02	*D*_x_ = 1.713 Mg m^−^^3^
Orthorhombic, *P**b**c**a*	Mo *K*α radiation, λ = 0.71073 Å
*a* = 12.3035 (7) Å	µ = 1.49 mm^−^^1^
*b* = 11.6560 (7) Å	*T* = 298 K
*c* = 20.7112 (10) Å	Block, blue
*V* = 2970.2 (3) Å^3^	0.25 × 0.20 × 0.20 mm
*Z* = 8	

### Data collection

**Table:**

Bruker SMART APEX CCD area detector diffractometer	3626 independent reflections
Radiation source: fine-focus sealed tube	3024 reflections with *I* > 2σ(*I*)
Graphite monochromator	*R*_int_ = 0.045
Detector resolution: 8 pixels mm^-1^	θ_max_ = 30.5°, θ_min_ = 2.0°
ω and φ scans	*h* = 0→17
Absorption correction: multi-scan (*SADABS*; Bruker, 2003)	*k* = 0→16
*T*_min_ = 0.707, *T*_max_ = 0.755	*l* = 0→19
11218 measured reflections	

### Refinement

**Table:**

Refinement on *F*^2^	Primary atom site location: structure-invariant direct methods
Least-squares matrix: full	Secondary atom site location: difference Fourier map
*R*[*F*^2^ > 2σ(*F*^2^)] = 0.039	Hydrogen site location: inferred from neighbouring sites
*wR*(*F*^2^) = 0.092	H atoms treated by a mixture of independent and constrained refinement
*S* = 1.07	*w* = 1/[σ^2^(*F*_o_^2^) + (0.0322*P*)^2^ + 3.2213*P*] where *P* = (*F*_o_^2^ + 2*F*_c_^2^)/3
3626 reflections	(Δ/σ)_max_ = 0.001
231 parameters	Δρ_max_ = 0.58 e Å^−^^3^
12 restraints	Δρ_min_ = −0.70 e Å^−^^3^

### Special details

**Table:**

Geometry. All e.s.d.'s (except the e.s.d. in the dihedral angle between two l.s. planes) are estimated using the full covariance matrix. The cell e.s.d.'s are taken into account individually in the estimation of e.s.d.'s in distances, angles and torsion angles; correlations between e.s.d.'s in cell parameters are only used when they are defined by crystal symmetry. An approximate (isotropic) treatment of cell e.s.d.'s is used for estimating e.s.d.'s involving l.s. planes.
Refinement. Refinement of *F*^2^ against all reflections. The weighted *R*-factor *wR* and goodness of fit *S* are based on *F*^2^, conventional *R*-factors *R* are based on *F*, with *F* set to zero for negative *F*^2^. The threshold expression of *F*^2^ > σ(*F*^2^) is used only for calculating *R*-factors(gt) *etc*. and is not relevant to the choice of reflections for refinement. *R*-factors based on *F*^2^ are statistically about twice as large as those based on *F*, and *R*- factors based on ALL data will be even larger.

### Fractional atomic coordinates and isotropic or equivalent isotropic displacement parameters (Å^2^)

**Table:**

	*x*	*y*	*z*	*U*_iso_*/*U*_eq_	
Ni1	0.05162 (2)	0.72886 (2)	0.655841 (16)	0.01042 (10)	
S1	−0.25226 (4)	0.98567 (4)	0.69683 (3)	0.01034 (14)	
O1	−0.10901 (13)	0.73405 (14)	0.62681 (9)	0.0138 (4)	
N1	0.07768 (16)	0.56644 (16)	0.61777 (11)	0.0128 (4)	
C1	0.0689 (2)	0.4662 (2)	0.64883 (14)	0.0162 (5)	
H1	0.0453	0.4665	0.6915	0.019*	
O2	0.20770 (14)	0.72708 (14)	0.69286 (9)	0.0133 (4)	
N2	0.09637 (16)	0.77000 (16)	0.56277 (11)	0.0134 (4)	
C2	0.0934 (2)	0.3618 (2)	0.62007 (14)	0.0184 (6)	
H2	0.0871	0.2937	0.6431	0.022*	
O3	−0.00380 (14)	0.67614 (14)	0.74609 (9)	0.0150 (4)	
C3	0.1271 (2)	0.3612 (2)	0.55698 (14)	0.0208 (6)	
H3	0.1440	0.2923	0.5366	0.025*	
O4	0.04198 (14)	0.89878 (14)	0.68080 (10)	0.0170 (4)	
C4	0.1357 (2)	0.4641 (2)	0.52378 (14)	0.0178 (5)	
H4	0.1576	0.4651	0.4808	0.021*	
O5	−0.25137 (14)	0.85952 (13)	0.70127 (9)	0.0172 (4)	
C5	0.11122 (18)	0.56556 (19)	0.55584 (13)	0.0128 (5)	
O6	−0.13912 (13)	1.02854 (14)	0.69611 (8)	0.0142 (4)	
C6	0.11779 (18)	0.67945 (19)	0.52374 (13)	0.0133 (5)	
O7	−0.30835 (14)	1.02193 (14)	0.63785 (9)	0.0157 (4)	
C7	0.1406 (2)	0.6931 (2)	0.45869 (14)	0.0185 (6)	
H7	0.1578	0.6300	0.4332	0.022*	
O8	−0.30946 (13)	1.03373 (13)	0.75434 (8)	0.0137 (4)	
C8	0.1376 (2)	0.8021 (2)	0.43219 (14)	0.0201 (6)	
H8	0.1508	0.8130	0.3884	0.024*	
C9	0.1145 (2)	0.8947 (2)	0.47183 (14)	0.0214 (6)	
H9	0.1122	0.9687	0.4552	0.026*	
C10	0.0950 (2)	0.8751 (2)	0.53642 (14)	0.0174 (6)	
H10	0.0801	0.9375	0.5629	0.021*	
H1A	−0.134 (3)	0.6650 (17)	0.6270 (18)	0.048 (11)*	
H2A	0.233 (3)	0.6601 (17)	0.7021 (14)	0.030 (9)*	
H3A	−0.0628 (19)	0.636 (3)	0.7451 (17)	0.042 (11)*	
H4A	0.092 (2)	0.937 (3)	0.7005 (15)	0.040 (10)*	
H1B	−0.152 (3)	0.778 (2)	0.6482 (16)	0.041 (11)*	
H2B	0.220 (3)	0.773 (2)	0.7246 (14)	0.048 (12)*	
H3B	0.046 (3)	0.639 (3)	0.766 (2)	0.071 (15)*	
H4B	−0.0191 (18)	0.934 (3)	0.6840 (17)	0.039 (10)*	

### Atomic displacement parameters (Å^2^)

**Table:**

	*U*^11^	*U*^22^	*U*^33^	*U*^12^	*U*^13^	*U*^23^
Ni1	0.00799 (13)	0.01272 (14)	0.0105 (3)	0.00003 (10)	0.00070 (11)	−0.00085 (11)
S1	0.0079 (2)	0.0117 (2)	0.0114 (4)	0.00052 (17)	0.0005 (2)	−0.0002 (2)
O1	0.0087 (7)	0.0155 (7)	0.0173 (12)	−0.0007 (6)	−0.0004 (7)	−0.0022 (7)
N1	0.0111 (9)	0.0163 (9)	0.0109 (15)	−0.0006 (7)	−0.0008 (8)	−0.0005 (8)
C1	0.0152 (11)	0.0184 (11)	0.0149 (18)	−0.0010 (9)	−0.0010 (9)	0.0009 (9)
O2	0.0111 (7)	0.0148 (7)	0.0139 (12)	0.0004 (6)	−0.0015 (7)	−0.0002 (7)
N2	0.0094 (8)	0.0162 (9)	0.0146 (14)	0.0007 (7)	0.0011 (8)	−0.0004 (8)
C2	0.0163 (11)	0.0163 (10)	0.0227 (19)	0.0002 (9)	−0.0029 (10)	0.0012 (10)
O3	0.0112 (8)	0.0205 (8)	0.0132 (12)	−0.0009 (6)	0.0008 (7)	0.0023 (7)
C3	0.0189 (12)	0.0195 (11)	0.024 (2)	0.0035 (9)	−0.0010 (10)	−0.0055 (10)
O4	0.0098 (8)	0.0171 (8)	0.0241 (13)	0.0013 (6)	−0.0022 (7)	−0.0062 (7)
C4	0.0171 (11)	0.0221 (11)	0.0143 (18)	0.0030 (9)	0.0027 (10)	−0.0046 (10)
O5	0.0198 (9)	0.0115 (7)	0.0204 (12)	0.0015 (6)	0.0071 (7)	0.0001 (7)
C5	0.0080 (9)	0.0179 (10)	0.0126 (17)	−0.0005 (8)	0.0000 (9)	−0.0010 (9)
O6	0.0083 (7)	0.0191 (8)	0.0153 (12)	−0.0020 (6)	0.0005 (6)	−0.0008 (7)
C6	0.0091 (9)	0.0172 (10)	0.0137 (17)	0.0006 (8)	0.0020 (8)	0.0011 (9)
O7	0.0159 (8)	0.0187 (8)	0.0125 (11)	0.0025 (6)	−0.0041 (7)	−0.0019 (7)
C7	0.0149 (11)	0.0250 (11)	0.0157 (19)	−0.0006 (9)	0.0013 (10)	−0.0012 (10)
O8	0.0115 (7)	0.0159 (7)	0.0137 (12)	−0.0003 (6)	0.0025 (7)	−0.0005 (6)
C8	0.0160 (11)	0.0304 (13)	0.0139 (18)	−0.0021 (10)	0.0033 (10)	0.0049 (11)
C9	0.0161 (11)	0.0243 (12)	0.024 (2)	−0.0006 (9)	0.0015 (10)	0.0105 (11)
C10	0.0154 (11)	0.0166 (10)	0.0201 (19)	0.0028 (9)	0.0053 (10)	0.0027 (10)

### Geometric parameters (Å, º)

**Table:**

Ni1—O4	2.0504 (17)	C2—C3	1.371 (4)
Ni1—N2	2.061 (2)	C2—H2	0.9300
Ni1—O1	2.0667 (17)	O3—H3A	0.864 (18)
Ni1—O2	2.0679 (17)	O3—H3B	0.862 (18)
Ni1—N1	2.0757 (19)	C3—C4	1.387 (4)
Ni1—O3	2.0824 (19)	C3—H3	0.9300
S1—O7	1.4653 (19)	O4—H4A	0.861 (18)
S1—O5	1.4732 (16)	O4—H4B	0.861 (18)
S1—O6	1.4791 (17)	C4—C5	1.390 (3)
S1—O8	1.4926 (18)	C4—H4	0.9300
O1—H1A	0.862 (18)	C5—C6	1.487 (3)
O1—H1B	0.862 (18)	C6—C7	1.386 (4)
N1—C1	1.338 (3)	C7—C8	1.384 (4)
N1—C5	1.347 (3)	C7—H7	0.9300
C1—C2	1.388 (3)	C8—C9	1.386 (4)
C1—H1	0.9300	C8—H8	0.9300
O2—H2A	0.862 (17)	C9—C10	1.378 (4)
O2—H2B	0.863 (18)	C9—H9	0.9300
N2—C10	1.341 (3)	C10—H10	0.9300
N2—C6	1.355 (3)		
			
O4—Ni1—N2	91.51 (8)	C6—N2—Ni1	115.37 (16)
O4—Ni1—O1	89.41 (7)	C3—C2—C1	118.7 (2)
N2—Ni1—O1	88.66 (8)	C3—C2—H2	120.7
O4—Ni1—O2	88.28 (7)	C1—C2—H2	120.7
N2—Ni1—O2	95.80 (8)	Ni1—O3—H3A	114 (2)
O1—Ni1—O2	175.03 (8)	Ni1—O3—H3B	111 (3)
O4—Ni1—N1	170.30 (8)	H3A—O3—H3B	109 (3)
N2—Ni1—N1	79.38 (8)	C2—C3—C4	119.4 (2)
O1—Ni1—N1	93.66 (7)	C2—C3—H3	120.3
O2—Ni1—N1	89.32 (7)	C4—C3—H3	120.3
O4—Ni1—O3	92.28 (8)	Ni1—O4—H4A	125 (2)
N2—Ni1—O3	174.58 (7)	Ni1—O4—H4B	122 (2)
O1—Ni1—O3	87.52 (7)	H4A—O4—H4B	110 (3)
O2—Ni1—O3	88.18 (7)	C3—C4—C5	118.9 (3)
N1—Ni1—O3	97.03 (8)	C3—C4—H4	120.6
O7—S1—O5	110.09 (10)	C5—C4—H4	120.6
O7—S1—O6	109.72 (10)	N1—C5—C4	121.9 (2)
O5—S1—O6	109.32 (10)	N1—C5—C6	115.8 (2)
O7—S1—O8	109.57 (10)	C4—C5—C6	122.3 (2)
O5—S1—O8	109.16 (10)	N2—C6—C7	122.0 (2)
O6—S1—O8	108.96 (10)	N2—C6—C5	114.7 (2)
Ni1—O1—H1A	108 (3)	C7—C6—C5	123.3 (2)
Ni1—O1—H1B	117 (3)	C8—C7—C6	119.1 (2)
H1A—O1—H1B	109 (3)	C8—C7—H7	120.5
C1—N1—C5	118.4 (2)	C6—C7—H7	120.5
C1—N1—Ni1	126.97 (18)	C7—C8—C9	119.0 (3)
C5—N1—Ni1	114.57 (15)	C7—C8—H8	120.5
N1—C1—C2	122.8 (3)	C9—C8—H8	120.5
N1—C1—H1	118.6	C10—C9—C8	118.8 (2)
C2—C1—H1	118.6	C10—C9—H9	120.6
Ni1—O2—H2A	115 (2)	C8—C9—H9	120.6
Ni1—O2—H2B	116 (3)	N2—C10—C9	123.0 (2)
H2A—O2—H2B	110 (3)	N2—C10—H10	118.5
C10—N2—C6	118.1 (2)	C9—C10—H10	118.5
C10—N2—Ni1	126.14 (18)		
			
O4—Ni1—N1—C1	158.1 (4)	C1—C2—C3—C4	0.0 (4)
N2—Ni1—N1—C1	178.5 (2)	C2—C3—C4—C5	0.8 (4)
O1—Ni1—N1—C1	−93.6 (2)	C1—N1—C5—C4	0.5 (3)
O2—Ni1—N1—C1	82.5 (2)	Ni1—N1—C5—C4	177.86 (18)
O3—Ni1—N1—C1	−5.6 (2)	C1—N1—C5—C6	179.2 (2)
O4—Ni1—N1—C5	−19.0 (6)	Ni1—N1—C5—C6	−3.5 (2)
N2—Ni1—N1—C5	1.39 (16)	C3—C4—C5—N1	−1.1 (4)
O1—Ni1—N1—C5	89.34 (16)	C3—C4—C5—C6	−179.7 (2)
O2—Ni1—N1—C5	−94.63 (16)	C10—N2—C6—C7	1.7 (3)
O3—Ni1—N1—C5	177.28 (16)	Ni1—N2—C6—C7	174.92 (19)
C5—N1—C1—C2	0.4 (3)	C10—N2—C6—C5	−176.5 (2)
Ni1—N1—C1—C2	−176.60 (18)	Ni1—N2—C6—C5	−3.2 (3)
O4—Ni1—N2—C10	−9.6 (2)	N1—C5—C6—N2	4.5 (3)
O1—Ni1—N2—C10	79.7 (2)	C4—C5—C6—N2	−176.9 (2)
O2—Ni1—N2—C10	−98.1 (2)	N1—C5—C6—C7	−173.6 (2)
N1—Ni1—N2—C10	173.7 (2)	C4—C5—C6—C7	5.0 (4)
O3—Ni1—N2—C10	124.8 (7)	N2—C6—C7—C8	−2.4 (4)
O4—Ni1—N2—C6	177.75 (17)	C5—C6—C7—C8	175.5 (2)
O1—Ni1—N2—C6	−92.87 (17)	C6—C7—C8—C9	1.6 (4)
O2—Ni1—N2—C6	89.33 (17)	C7—C8—C9—C10	−0.2 (4)
N1—Ni1—N2—C6	1.11 (16)	C6—N2—C10—C9	−0.1 (4)
O3—Ni1—N2—C6	−47.8 (8)	Ni1—N2—C10—C9	−172.58 (19)
N1—C1—C2—C3	−0.7 (4)	C8—C9—C10—N2	−0.6 (4)

### Hydrogen-bond geometry (Å, º)

**Table:**

*D*—H···*A*	*D*—H	H···*A*	*D*···*A*	*D*—H···*A*
O1—H1*B*···O5	0.86 (2)	1.90 (2)	2.754 (2)	173 (3)
O1—H1*A*···O7^i^	0.86 (2)	1.83 (2)	2.683 (2)	173 (4)
O2—H2*B*···O5^ii^	0.86 (2)	1.87 (2)	2.728 (2)	174 (3)
O2—H2*A*···O8^iii^	0.86 (2)	1.97 (2)	2.800 (2)	162 (3)
O3—H3*B*···O6^iii^	0.86 (2)	1.89 (2)	2.736 (2)	168 (4)
O3—H3*A*···O8^i^	0.86 (2)	1.98 (2)	2.840 (2)	172 (3)
O4—H4*B*···O6	0.86 (2)	1.86 (2)	2.712 (2)	172 (3)
O4—H4*A*···O8^ii^	0.86 (2)	1.90 (2)	2.760 (2)	174 (3)
C8—H8···O6^iv^	0.93	2.55	3.310 (3)	139

Symmetry codes: (i) −*x*−1/2, *y*−1/2, *z*; (ii) *x*+1/2, *y*, −*z*+3/2; (iii) −*x*, *y*−1/2, −*z*+3/2; (iv) −*x*, −*y*+2, −*z*+1.
